# Abstracts and Gleanings

**Published:** 1888-08

**Authors:** 


### ABSTRACTS AND GLEANINGS.

**The Treatment of Tymphanites by Puncture.**—At the meeting of the Hunterian Society, held March 28, 1888. Dr. R.J. Ryle read a paper on a case of tympanites treated by puncture thro gh the abdom nal wall (*Lancet*, April 7, 1888). The patient, a man aged 46, came under treatment in October, 1887, with vomiting, paroxysmal pain, and constipation of about ten days' duration. Examination of the abdomen showed considerable fulness, but no tumor was felt, and examination per rectum revealed nothing. Enemata and O'Beirne's tube gave no information. Colotomy was proposed, but declined by the patient; therefore puncture of the intestine to relieve distension was resorted to as the only treatment which promised relief. Eight punctures were made, with considerable relief to the distension, but with the result of setting up an attack of violent peristaltic contraction about half an hour afterwards. Three days later it was necessary to puncture again in three places, and two precautions were used to prevent peristalsis, viz.: (1) Hypodermic injection of morpho-atropine, and (2) use of a larger aspirating needle. A very large quantity of gas was evacuated. No violent peristalsis followed. Forty-eight hours later the bowels opened. After a fortnight symptoms of obstruction returned, and puncture again became necessary, the same precautions being taken as on the former occasion, and in the course of the following three weeks puncturing was four times repeated. Great temporary relief was gained, but the patient gradually sank and died. A post mortem examination showed an annular stricture of the first part of the rectum, but no evidence of peritonitis or extravasation of fæces, and with the exception of certain small black spots no trace of the puncture was visible in the wall of the bowel. The points of interest in the diagnosis of this case of chronic obstruction were then discussed; and, secondly, those of the treatment by puncture. As to the latter, the relief afforded by this procedure was of value for three reasons: 1. As checking the respiratory and circulatory difficulties consequent on extreme distension. 2. As diminishing the direct risks of distension—viz., rupture and peritonitis. 3. As favoring the re-establishment of the normal intestinal action. The chief mode, however, in which puncture tended to produce evacuation of the bowels is by exciting brisk peristalsis, and this is a result which, as in the present instance, may require to be guarded against. As to the dangers of intestinal puncture, it is impossible to state that there are none; thinning or ulceration of some part of the bowel may convert a puncture into a rent. The same might happen from movement of the empaled bowel, or liquid fæces might be conveyed from the needle to the peritoneum. But a much less hypothetical danger seemed to be the rupture of the diseased bowel by the violent peristalsis set up by puncturing, or if the puncturing is very free. The reality of this risk is shown (possibly) by the history of a case of Dr. Bristowe's, recorded in the Pathological Society Transactions for 1872; and still more clearly by a case recorded in 1878 in the *British Medical Journal* by Dr. Coupland and Mr. Morris. in which rupture was due to peristalsis caused by puncture. If this view of the danger be corract, the proceeding comes to resemble the administration of ergot during labor, and the cases most suitable for the treatment would seem to be those which are known to be characterized by healthy bowel and an abscence of insuperable obstruction, such as sometimes occurs in obstetric practice or after abdominal operations. Perhaps, too, as has been suggested by Dr. Galabin, this treatment might be of use in commencing peritonitis. The beneficial effects of simple relief of tension in checking the progress of inflammation are shown in periostitis and cellulitis, and probably a few needle punctures would be less dangerous than continued distension to the peritoneal coat. From similar considerations he suggested its employment in enteric fever, more especially, if, as was probable, excessive peristalsis can be prevented by opium. The specimen was shown at the meeting.—*Therapeutic Gazette.*

**Chloride of Sodium in the Sickness of Pregnancy.**—W. T. Greene, M. D., (*Medical Press*, March 7), says: I have recently had two very severe cases of sickness during pregnancy, the first patient being reduced in spite of all kinds of remedies, and a consultation with an eminent obstetrician, to such an extent that I thought the induction of premature labor would be the only means of saving her life. It was her seventh pregnancy, and she was never similarly affected before, nor can I account in any way for the severity of her symptoms. After trying everything I could think of without effect, I chanced to remember that I had found small doses of chloride of sodium (common salt) in chloroform water very effective in arresting infantile sickness, and concluded to give it a trial, and did so. The effect was really magical. After the first dose, 5 grains in 1 ounce of chloroform water, the sickness was lessened, and by the time she had taken six doses it had quite left her, and she was able to take and retain solid food, which it had been impossible for her to do for months previously. I found it necessary, however, to continue the medicine, though at more distant intervals, three times a day, for when it was left off for a few days the sickness returned. This patient went on favorably, had a good labor, and made a good recovery. In another case the patient was so reduced by the continual vomiting, that I feared an attack of puerperal maniia from sheer anæmia, as she was in a most desponding state. I tried the chloride of sodium with the same beneficial result as in the previous case, and this patient also made an excellent recovery. In the case of both patients the secretions were very acid, yet soda, potash, and ammonia had no effect, or at least only a very fugitive effect upon the symptoms.—*New York Medical Abstract.*

**Treatment of Heat Fever or Sunstroke.**—Dr. F. A. Packard, resident physician in Pennsylvania Hospital, in a summary of an article in the *American Journal of Medical Sciences*, says: As soon as a patient with *sun stroke* or *heat fever* is brought to the hospital, he is stripped at once and placed on a water-proof fracture bed, and a thermometer inserted into the rectum. (The thermometer always registers from 106° in mild, to 112° in extreme cases.) Ice is at once packed around the body and extremities, and usually at the very outset, 15 or 20 minims of tr. digitalis is administered hypodermatically. The thermometer is removed every seven minutes, and the ice packing continued until the rectal temperature falls to 104°. The patient is then dried and put to bed, and an ice cap applied to the head. If the ice packing be kept up after the rectal temperature has fallen below 104°, there is apt to be a too rapid and great fall, and external heat and free stimulation will be required.

The above is the mode of treatment used in cases where the temperature exceeds 105 2-5°. In all cases with a temperature below 106 2-5 degress, the body having been stripped, is sponged freely with a solution of one part of alcohol and four parts of ice water, an ice cap being applied at the same time to the head. If convulsions follow, after a considerable reduction of temperature, morphia hypodermatically will have a good effect. If the pulse and respiration do not improve in character after the temperature has fallen, bleeding will a most invariably be followed by quieter breathing and a soft regular pulse. In this case a feeble pulse does not contra-indicate bleeding. If the face is congested or livid, the capillary circultion engorged or obstructed, the heart's action labored, the breathing shallow and stertorous, and the pupils contracted, resort was always, first to wet cups behind the ears. In the majority of cases only a few drops of *black altered blood* could thus be abstracted. In no case, when cupping was tried, could enough blood be removed to affect the general or cerebral circulation. Bleeding from the basilic vein, in case it is deemed necessary, should be employed, and even from this free outlet the blood will often flow only by being squeezed up from the hand, and then it issus in black, thick jets. In case of bleeding, twelve or sixteen ounces will generally suffice to produce a decided improvement in circulation, color and respiration. The pulse is almost invariably absent from the wrist in cases where the temperature is over 108°. It will usually take only from fifteen minutes to a half hour, to reduce the temperature by means of the ice pack.—*Medical Waif.*

**Clinical Morphologies**—At the late meeting of the American Medical Association, a correspondent of *Albany Medical Annals*, writes: My own particular work was to assist Dr. Cutter at this meeting in his demonstration by the lantern of plates on the clinical morphologies. The plates used did not by any means cover the subject; but, for that matter, when will any science be actually complete that has anything to do with medicine?

The demostrations were: The blood in health and in pretuberculosis, rheumatism, fibræmia, thrombosis, embolism, syphilis, eczema, and fibrous consumption; the morphology of the sputum in tuberculosis, asthma, diphtheria, croup and in other conditions which cannot be named, as the nomenclature of medicine to-day is not exact.

One plate which attracted much attention was made from a direct photograph of diphtheria spores. Dr. Cutter said that some ten years ago he removed some diphtheria membrane from the uvula of his daughter, dead of this awful disease, though he had done all he could, even performing tracheotomy. He put the specimen in a bottle of pure carbolic acid, and three and a half years later, having a room in which he could project by sunlight on a screen, he prepared a specimen from this membrane, and found that the spores were still living He said he considered it was about time to stop, as he did not want to risk his life, and so took the photograph and closed his investigations.

This is very strong evidence that we must not confine our studies to the action of germicides on bacteria, micrococci and bacilli, but to the higher forms of microbian life, as the spores and mycelial filaments.

It would also seem that to America will be given the honor of working out many of these phases of morphology; for as far as I can find out, Salisbury antedated Koch's discovery of the bacillus many years, but did not stop with this baby form, but so worked up the disease by synthesis and analysis that to-day phthisis is not only a curable disease, but may be diagnosticated by the blood morphology from six to twelve months before the lung tissues are invaded. This is only a statement of my actual daily medical work, and is given here as an impression on the minds of others at this meeting.

The other morphologies illustrated were of the fæces, urine, skin and foods. In the last, attention was directed to infants' foods, so-called, flours, drinking waters, various vegetables, etc.

These plates were made either from microphotographs (some taken with the 1-50th and 1-75th inch objectives) or from micrographical drawings.

**Agaricine.**—Dr. Alex. B. Pope, of New York, writes the *Therapeutic Gazette*: In reply to the request published in the *Therapeutic Gazette* of April 16, 1888, on the subject of agaricine, it gives me much pleasure to offer the results of my experience while on service in Bellevue Hospital, in order that those of the profession who have not used it may be induced to test what I consider one of our most useful therapeutic agents. At the suggestion of my visiting physician, Dr. George M. Tuttle, an investigation into the merits of agaricine in controlling the night sweats of phthisis was undertaken, and with most gratifying results.

It was prescribed in doses of 1/8 of a grain at bed time, and repeated usually twice during the night at intervals of two, three, or four hours. In the majority of cases this was sufficient to completely control the night sweating. In other cases it was given every four hours, day and night, so that six doses of 1/8 of a grain each were taken. I remember but one case in which the drug was pushed beyond this limit, because of failure in doses of 1/8 of a grain every four hours, and in this instance 1½ grain were given in divided doses during the twenty-four hours, and failing again, it was discontinued because of s.mpto.s which were believed to be due to the drug. Unfortunately, I cannot give the details of the disturbances which were attributed to it, but, if my memory is not at fault, they were gastro intestinal and nervous, the latter affecting especially the ce ebrum and sensory nerves.

In several instances agaricine succeeded when belladonna or atropine failed, and without giving rise to any unpleasant symptoms whatever.

The failure of atropine in some instances was due to the fact that it was not well borne, on account of great dryness of the mouth or nervous excitement occasioned by it.

My conclusions and convictions were drawn from a series of cases, probably over one hundred, of advanced diseases, such as one sees in the wards of a public institution. Most of them were treated by agaricine alone,—*i.e.*, without the administation of any other drug which could affect the symptoms under consideration.

After having used this drug in this manner for some time, and having convinced ourselves of its value, we used a combination of agaricine and aromatic sulphuric acid, in order to effect solution and get the benefit of each of these remedies.

The formula used is as follows:

℞. Agaricini.................................gr. x,
 Acidi sulphuric aromat.......................*m* mcc.

Dissolve and filter. Dose, 10 minims in syrup or simple elixir, containing 1-12 grain of agaricine, at night only, or every four hours.

If desirable, atropine, gr. 1, may be added to the prescription above; and if this combination, pushed if necessary, does not control sweating, I know of nothing that will.

Not only is agaricine useful in the night sweats of phthisis, but sweating from other causes, as debility after the use of antipyrin, antifebrin, or other antipyretics.

The agaricine used was the pure white agaricine of musk,—properly speaking, agaric acid.

I am much indebted to Professor Charles Rice for valuable assistance in prosecuting this study.

**Another Triumph of Laboratory Research—Tasteless Preparations of Sagrada** (*Rhamnus Purshiana*).—Abstract of an article entitled "An Examination of Cascara Sagrada," by H. F. Meier and J. Leroy Webber, published in the *American Journal of Pharmacy*, February, 1888.

Recent investigation of the constituents of cascara sagrada has led to the discovery of new principles and facts of great importance pharmaceutically and therapeutically.

The chief objection to cascara sagrada heretofore has been its inherent bitterness. In the light of recent researches, tasteless preparations of this drug highly efficacious medicinally are now to be had.

These discoveries mark a distinct advance in pharmacal attainment and in the therapeutics of chronic constipation, since this remedy can now be much more generally and persistently administered, and its well-known tonic laxative action obtained from its employment.

The facts disclosed concerning this remedy deserve more than a passing notice, especially since they indicate the existence of principles and modes of action extending far beyond the subject indicated, and are well worth the close attention of the thoughtful and scientific physician. A valuable contribution to the knowledge of the chemical constitution of this drug appeared in the *American Journal of Pharmacy*, for Febuary, 1888, which makes it possible not only to obtain a true interpretation of the various clinical observations, but clears up apparent anomalies and also indicates the reason for observed effects, which have lately been disputed, but now admit of no further question or misunderstanding.

Among the discoveries referred to in this valuable paper, of especial interest to the physician, is the influence of a class of vegetable ferments and their recognition as the causes of various abnormal conditions, such as colic, vomiting, nausea, diarrhœa and dysentery, which occasionally attend the administration of certain drugs.

It appears that frangula bark, when fresh, contains such a ferment in excessive quantities and is, therefore, unfit for the use until the ferment has exhausted itself—the process usually occupying several years. It also appears that cascara sagrada contains some of this principle, and this fact will account for the occasional untoward effects of the drug, which have been observed as consequent on the employment of a number of its preparations heretofore in the market. These effects are, therefore, not due, as has been supposed, to any idiosyncrasy on the part of the patient, or to the laxative or tonic constituents of the bark itself, but to a distinct objectionable principle, which once recognized can be rendered inoperative and harmless.

It has been reserved for Parke, Davis & Co., through their exhaustive investigations, to be the first to clearly recognize the principles involved, and by the application of such intelligent comprehension, to formulate and adopt correct pharmaceutical processes and thus overcome all the difficulties heretofore existing. As a result of their investigations, they now offer to the medical profession a fluid extract, a solid extract and also a concentration, all of which (designated as "formula of 1887") exhibit only the desirable laxative and tonic properties, and being free from this ferment, are incapable of producing griping, nausea or any of the mal effects above enumerated.

It appears that these ferments are distributed through a large number of vegetable substances, being not confined to unripe fruits only, but can also exist in the root, bark, leaf, or even in vegetable extracts of which we have illustrations in various juices, liquid or inspissated. Of this latter class a oes will serve for an example. A familiar illustration of an unaltered vegetable would be the cucumber, the green apple (familiar to the school boy), and unripe fruit generally. In the case of the cucumber, experience has taught the means of removing this ferment by dialysis or osmosis. We spr nkle salt over it or surround it with a strong brine which provokes an outward flow of the fluid containing the ferment, with the result that the ferment is to a large extent removed, and thus rendered incapable of producing the same conditions in the stomach, for which it was intended in the plant; that is, the creation of vegetable acids from other material previously existing, in the same manner that pepsin, likewise an unorganized and soluble ferment, provokes the solution of fibrin and albumen, forming peptone, or as diastase is capable of effecting the transformation of starch into soluble glucose and dextrin, both new bodies.

That these ferments all bear a direct quantitative proportion to the results accomplished, has been practically recognized. We are promised a satisfactory indication of the sources of the acids formed in the plant, which will enable us to corroborate the statements that identical processes go on in the stomach when the ferment is permitted to exert its action there.

The physiological tests now being conducted at the laboratory of Parke, Davis & Co., with the different principles contained in the plant, cannot fail to demonstrate finally not only the superioiity of cascara itself, to its former supposed competitor frangula, but also its comparative value as a laxative.

To physicians desiring fuller information concerning the discoveries made, a reprint of the article from the *American Journal of Pharmacy* and a working bulletin descriptive of this drug will be mailed by Parke, Davis & Co., free on request.

**Poisonirg with Cocaine.**—The London *Lancet*, of May 5, 1888, has been received, and we find in it an account by Surgeon A. W. Addinsell of his own experience with cocaine. One grain of the hydrochlorate dissolved in 20 minims of water was injected into a small abscess at the root of the tooth. In three minutes the pain of the abscess ceased, and the tooth was extracted without pain. Directly afterwards Mr. Addinsell says, "I then began to feel giddy and sick. I felt my pulse; was very rapid and weak. I had violent palpitation of the heart, with a great thumping at the chest-wall, and a slight feeling of suffocation. I then became very talkative and laughed loudly. I remember insisting upon Mr. Maitland (who had extracted the tooth) getting into his own operating-chair, and my wanting to opeiate; this was politely but firmly declined. I now felt very sick, a d tingled all over. I staggered about the room, was very pale, and my pupils were widely dilated. I was perfectly conscious, however. I remained in this condition for thirty-five minutes, when the pain in the gum began gradually to return, my pulse improved, and in an hour after the injection I returnd home."

In a case of a lady who also was suffering from a periodontic abscess, Mr. Addinsell injected 1 grain. In two minutes the patient began to laugh and walk about the room excitedly. Suddenly she fe'l into a chair, and cried out, "Oh, my legs; they feel so strange!" She became very pallid, with exceedingly feeb e, rapid pulse, and complained of tingling all over, and burning pain at the stomach. Her eyes were staring and dazed-looking, pupils dilated, and cold perspiration suffused her ashy-pale face. Under the influence of brandy, hot external applications, etc.; she revived somewhat, vomited, and in one hour from the time of injection fell into a quiet sleep.—*Therapeutic Gazette.*

**Peppermint Water in Pruritus Pudendi**—Dr. Amand Routh calls attention to the value of peppermint water in pruritus pudendi. In pruritus due to pediculi, ascarides, an irritable urethral caruncle, an endocervical polypus, early cancer of the cervix, distension of Bartholini's ducts or glands, the leucorrhœa of vaginitis, endocervicitis, and metritis, or the irritating discharges of advanced carcinoma uteri, or to a gouty or diabetic diathesis, peppermint water excels all others, cocaine inclusive, in affording relief, whilst endeavors are being made to remove the cause. The agent here alluded to is peppermint water used as a lotion. The B. P. preparation answers well, but is bulky for carrying about, and is incapable of concentration unless rendered alkaline. This is best done by borax, as being in itself soothing and antiseptic. Patients can easily make their own lotion, as required for use, by putting a teaspoonful of borax into a pint bottle of hot water and adding to it 5 drops of oleum menthæ piperitæ and shaking well; the parts affected to be freely bathed with a soft sponge. If no cracks or sores are present, this lotion will remove the itching, but if there be eczema or a rawness from scratching, it is inapplicable. Olive oil, with five grs. of iodoform to the ounce, is then more useful. The greatest and most permanent relief is afforded in the neurosal form, especially in the pruritus which often accompanies pregnancy. It is also very useful in the pruritus which occurs at the climacteric, or in elderly women, in whom it may be only part of a general pruritus, and also in those cases of women of all ages where the urine simultaneously becomes of very low specific gravity without any evidence of having a gouty or granular kidney as a remote cause.—*British Medical Journal.*

**A Case of Mammary Cancer Treated by Inoculation with Erysipelas**—Axel Holst reports (*Centralblatt fur Bakteriologie und Parasitendunde*, Number 13, 1888) the following: A woman, forty years of age, otherwise in good health and well-nourished, had a scirrhus of the right breast one year ago which was then extirpated. A few months later the cicatrix became nodulated, and ulceration (skin cancer) appeared. In Aug., 1887, almost the entire surface of the right side of the chest, from the clavicle to the borders of the ribs and from the sternum to the posterio axillary line was involved, and the glands over the clavicle were large and hard, the axillary glands could not be felt through the infiltrated granulating tissue which here and there formed distinct tumors. As an ultimate resource the patient was inoculated with a culture of the erysipelatous coccus (the fifteenth generation from a case of erysipelas of the legs, obtained nineteen and a half months previously); a second inocculation was required before a result was obtained. Twenty-one hours after the last injection, the patient had a chill, and shortly afterward, an erysipelatous blush appeared around the ulcer and soon spread to the arm which presented the typical appearances of erysipelas. Seven days after the inoculation there was a sudden fall of the temperature, and the patient rapidly improved.

The result within the first ten months and a half was very striking; the upper surface began to cicatrize, the i filtration was much reduced. so that the disease appeared to be mo e superficial than before and the entire growth seemed to contract. The arm, however, remained swollen, and did not entirely recover from the ery·sipe as. Three months later, the skin again began to break down, the gland were enlarged, and the left breast became involved. The general health rapidly declined, and her strength was materially reduced.

While the ultimate result in this case was not encouraging, the reaction from the inoculation was accompanied by a period of inactivity of the cancer-growth during which repair took place. The immediate growth was very suggestive. In a case reported some years ago by Neelsen (*Centralblatt, fur Chirurgie*, 44, 84), rapid *increase* of the mammary cancer occurred after acc dental inoculation with erysipelas.—*Medical Times.*

**Napthol as an Antiseptic Medicament.**—Kaposi, several years ago, called attention to the value of this agent in the treatment of certain d seases of the skin, and particularly of scabies. He employs napthol in a ten per cent. alcoholic solution, or in the form of ointment, and says that one or two applications will cure the most inveterate cases of common itch. For this purpose an ointment may be made with one part of napthol and ten of vaseline. He has had equal success with naphthol in psoriasis, eczema, and ichthyosis, and there is nothing better, he says, to allay tormenting itching of prurigo.

Hensinger also speaks in high praise of naphthol as an external agent (alcoholic lotion or pomade) in the skin diseases mentioned above, in lupus erythematosus and in chancres. Under its use sores of a phagedenic character, rapidly take on a healthy action. Van Harlingen, moreover, has seen favorable results from naphthol in obstinate cutaneous affections.

The experiments to which allusion has been made, have led Bouchard to regard naphthol as one of the best and safest antiseptics. According to these a three per cent. solution markedly retards the development of the typhoid bacillus, as well as that of the bacillus tuberculosis. The same solution is atal to the microbes of several of the parasitic diseases of animals, and prevents fermentations. Bouchard gives naphthol internally in typhoid fever, and believes that, if it does not abort this disease, it certainly renders its course milder. He regards a dose of 45 grains (2 50 grammes) a day as realizing the conditions of intestial antisepsis, and affirms that in such doses there is no antiseptic agent which is more innocuous. This use of naphthol in typhoid fever has been followed to some extent in this country with apparently favorable results.—*Boston Medical and Surgical Journal.*

**A Cure of Tuberculosis.**—Prof. Luton, of Reims, in a recent long article, concludes that the cure of tuberculosis can generally be obtained by means of the phosphate of copper, which, however, must be in the nascent state and soluble in an alkaline body. For twenty-five years Dr. Luton has sought for this cure, and he thinks he has found a specific. He employs the following formula:

℞. Neutral acetate of copper....................gr 0.15,
Crystallized phosphate of sodium..............gr. 0 75,
Glycerin.................................
Powdered licorice..........................aa a. s. q.

This is for one pill.

A double decomposition takes place in the stomach, and there is a specific action on the part of the copper, with a dynamic action on the part of the phosphorus in the preparation.—*N. Y. Medical Journal.*

[The formula as given above seems liable to lead to error, and the dose recommended (6 to 8 pills daily) appears large. The following would appear to be more practicable and at least safer with which to make a trial of the copper.

℞. Neutral acetate of copper....................grs. iij,
Crystallized phosphate of sodium..............grs. xv,
Glycerin.................................
Powdered licorice..........................aa a. s. q.

This for forty eight pills, one after meals. The dose may be increased, if well borne.—*Eds. Journal Dietetics.*

**Experience in the Use of Cocaine.**—Dr. Edmunds says that on applying cocaine subcutaneously for the production of local anesthesia, it is not advisable to use a stronger solution than five per cent. In his earlier cases, in which this strength was used, constitutional symptoms were never seen; but when owing to the anethesia in one case not being sufficient, he used stronger solutions, there occasionally occurred one or more of the following symptoms: Pulse becoming very rapid, weak, and almost imperceptible; sense of faintness and feeling of distress in the region of the heart, blueness of lips and cold perspirations, restlessness, amounting almost to convulsive movements, and dilated pupils. Happily these symptoms never lasted very long; but as nothing of this sort was seen with a five per cent solution, it seems better not to go beyond that strength. Cocaine will entirely prevent the pain of the injection of tincture of iodine into the tunica vaginalis for the cure of hydrocele In two cases a solution of 5 grs. of cocaine in fifty minims of water, was injected through the canula after the fluid had been drawn off. When, after the lapse of five minutes, tincture of iodine was injected, there was no pain or feeling of faintness, nor was there any constitutional symptom visible from the cocaine. The iodine and the iodide of potassium in the tincture of iodine react with the cocaine chemically, but these changes do not prevent the cure of the hydrocele. It is true that the injection of a saturated solution of carbolic acid in glycerine into a hydrocele sac does not cause pain; but this treatment is apt to fail.—*Lancet.*—*Indiana Medical Journal.*

**Abdominal Surgery.**—In cases of removal of the ovaries, Montgomery prefers braided silk ligatures for ligating the pedicle, as he is then certain that the ligature will remain on long enough to avoid all danger of hemorrhage.

In the course of over forty operations of this character, he has had no untoward result from the presence of the ligature.

For sewing up the abdominal incision he uses silk gut. Two small needles are put on each suture, one at either end. Each needle is then passed from within out, care being taken that the peritoneum is included well within the suture.—*Medical Times.*

**Acid Indigestion.**—With great acidity of the stomach, there is generally a burning pain along the line of the œsophagus. Patients frequently complain of "heart-burn," too. For digestive trouble in a girl of ten, from acidity, he gave:

℞. Spiriti ammoniæ aromatici ........................ ʒ ij,

Sodii bicarbonatis ........................ ʒ i,

Syrupi ........................ ℥ i,

Aquæ ........................ ℥ iij.

M. Sig.—A dessertspoonful every three hours.

If there should be much pain in the stomach, he advised the mother to apply flannel wrung out of hot water.—*Medical Times.*

**Paraldehyde in Obstinate Vomiting.**—Dr. U. B. LaMoure, M.D., in *Albany Medical Annals*, says: Having been in the habit of prescribing, in my practice, paraldehyde in the treatment of insomnia in alcoholism, the patient usually being affected with gastritis, accompanied with obstinate vomiting. I have noticed that the first dose was sometimes rejected from the stomach, but the second, given usually in one or two hours, was almost invariably retained, notwithstanding the fact that for hours previous to treatment, in the majority of cases, not the lightest form of food or liquid would remain.

It occurred to me that the same remedy might be serviceable in checking vomiting in other cases. I have used it in ovarian irritability with sympathetic stomach disorder, in vomiting of pregnancy, and in the distressing nausea of migraine, with the most gratifying results. The formula employed is as follows:

℞. Paraldehyde ........................ *m* xl,
Elix. simp ........................ ℥ i.

M. S. One teaspoonful in a little water, repeated in half an hour, if required. This small dose in its effects is not hypnotic, acts as a sedative not only upon the mucous membrane of the stomach, but also has a tranquilizing effect upon the whole system. But few doses are usually required. The only objection to its use is its disagreeable odor.

**Kephir and its Use as an Infant Food.**—Dr. Longstreet Taylor has found kephir useful in feeding marasmic and hand-reared infants. He considers that it is superior to peptonized preparations and koumiss. The former is theoretically perfect, but practically not always satisfactory, either because the process is carried on too far or because injurious ptomaines are introduced during the process of preparation. The latter is too expensive and too difficult to obtain ever to be of much general service. Kephir is a product of the fermentation of cow's milk by which the thick coagulum which would otherwise form is broken up into fine flocculent flakes, which are much more amenable to the influence of the gastric juice. It must not be used after it has become too old and sour, as in this state it is neither palatable nor digestible. For young infants only the weak or young kephir in the first stage of fermentation should be used. It should be given luke-warm, *i. e.*, about 75° F. If the infant is less than a month old it may be diluted with one-third of water, the proportion of water being gradually diminished so that when the infant is six weeks old it may take the kephir undiluted.—*Archieves of Pediatrics*, May, 1888.

**Intra-Vascular Injections in Collapse.**—Dr. Diarkonoff has made a number of experiments on ten healthy dogs with a view of testing the effect of an intra-vascular injection of common salt, to which a little alkali has been added, on a collapsed condition of the organism. In order to induce collapse, chloroform was administered, and when the animal had shown signs of being in a more or less dangerous condition a small quantity of Schwarz's solution (which consists of chloride of sodium 6 grammes, caustic soda .05 gramme, in 1000 grammes of distilled water) was introduced into the distal end of the femoral artery, or into the proximal end of the femoral vein or the juglar vein. A remarkable effect was produced by these injections on the character of the pulse and on the blood-pressure. In some cases in which the heart appeared to have stopped, contractions were by this means reinduced. The effect on the respiration was not so constant; still, this usually improved. Of course, when the chloroform was pushed beyond a certain point, saline injection and artificial respiration alike failed.—*Lancet.*

**Thymus Vulgaris in the Treatment of Whooping-Cough.**—Dr. J. B. Johnson, of Washington, D. C., says, in the *Medical and Surgical Reporter*, March 17: I direct an ounce of common thyme to be put into one pint and a half of hot water, and boiled down to one pint, then strained and sweetened well with either honey or sugar. In this manner it is made pleasant to the child. To infants I order one or two teaspoonfuls to be given regularly every hour or two, and to children a tablespoonful every hour or two during the continuance of the disease. Should auscultation reveal much inflammatory action of the bronchial tubes or lungs, I usually add two drachms each of iodide of potassium and powdered chlorate of potash to each pint of the sweetened decoction, and direct it to be used in the same manner as the simple decoction. Under the influence of this treatment my little patients pass through attacks of whooping-cough with astonishing ease and comfort, and only on rare occasions have I been compelled to use any other medicines. This treatment is greatly aided by a prudent attention to diet, which should consist of milk and farinaceous foods.—*Pittsburgh Medical Review.*

**Treatment of Penetrating Gun-Shot Wounds of the Cranium.**—Dr. Joseph D. Bryant, in a paper read before the Medical Society of the State of New York (*New York Medical Journal*) concludes that:

1. A bullet should be removed from the brain as soon after its reception as its situation can be determined and the patient's condition will permit.
2. A bullet should be removed from the brain at a later period if symptoms supervene and it can be located.
3. A bullet that has been located in the brain and has not been removed, should be removed before the supervention of symptoms, when it assumes a migratory character.
4. No effort should be made to remove a ball from the brain if it cannot be located.—*News and Miscellany.*

**The New York Colleges.**—The New York medical schools, no doubt, have many advantages. But from a recent communication in the *Medical Record* by a student at one of the colleges, it would seem they have their disadvantages as well. He states that the lecture rooms are so crowded that it is almost impossible to obtain seats, and having procured his dissection ticket he waited for months for an opportunity to dissect, but at last was compelled to go to a Homœopathic college to get to dissect.

This may all be very well in didactic teaching, but clinical instruction under such difficulties is almost impossible.—*Progress.*

**Impure Antipyrin.**—The extraordinary demand for antipyrin is very much in excess of the supply, and great pressure is put upon the manufacturers to increase the amount of the manufactured article in the market. The consequence is that due care has not been shown in the purification of a drug, a certain proportion of benzine having been detected in samples submitted to analysis, according to Dr. Dujardin-Beaumetz. This impurity may account for some of the toxic symptoms which have been reported, such as cutaneous eruptions, gastric troubles and even grave cerebral symptoms, more particularly in the aged.—*British Medical Journal.*

**Strychnine in Alcoholism.**—According to recent experiments, strychnine undoubtedly neutralizes the intoxicating and narcotic effects of alcohol. It enables large quantities of alcohol.

to be taken for a considerable stretch of time without causing the usual organic lesions which follow the use of alcohol alone. Therapeutically, strychnine should be used in all forms of alcoholism; it may be regarded as a powerful prophylactic against alcoholism.—*Hospital Gazette.*

**Man's Power of Imagination.**—The power of imagination is supposed to be stronger in women than in men, but this was not shown in a recent hospital experiment. Dr. Durand, wishing to test the practical effect of mind disease, gave a hundred patients a dose of sweetened water. Fifteen minutes after entering apparently in great excitement, he announced that he had by mistake given a powerful emetic and preparations must be made accordingly. Eighty out of the hundred patients became thoroughly ill and exhibited the usual result of an emetic; twenty were unaffected. The curious part of it is, that with very few exceptions the eighty "emeticised" subjects were men, while the strong-minded few, who were not to be caught with chaff, were women.—*New Orleans Picayune.*

**Effects of Atropia on the Suckling Child.**—Atropia, as has been previously observed by Preyer, passes very quickly through the mother's circulation and effects the child almost simultaneously. This has been observed where a one per cent. solution was hypodermatically administered. In the use of this drug, even as with opium, morphia and chloral, it is necessary to understand the idiosyncrasises of different individuals, and use the greatest care to avoid the cumulative effect of the drug.—*Berlin Woch.*

**Veratrum Viride.**—Dr. Byrd, in *Medical and Surgical Reporter*, says: The efficacy of veratrum in puerperal fever has, in recent years, been brought to the notice of the profession. I will add here, however, that the reflex influence upon the brain, and the detergent effect upon the circulatory system, of croton oil, administered in 2 drop doses until the bowels respond well to its action, greatly assists veratrum in controlling puerperal convulsions.

**Tonsillitis.**—Let the patient wet his forefinger and dip it into powdered bicarbonate of sodium. The surface of the tonsil should be rubbed with the end of his finger every five minutes during half an hour, afterwards every hour during the same day. Three applications a day are then sufficient. The author since adopting this treatment has not had to lance a single inflamed tonsil.—*Lyon Medical.*

During the last year Dr. Hartamann (*British Medical Journal*) has treated otitis with installations of several drops of a solution (one in ten) of carbolized glycerine with excellent results. Pain instantly disappeared, and the progress of the affection was checked. In cases where effusion existed, the relief obtained was equally great. M. Rohrer, who confirms M. Hartamann's statements, recommends a solution of twenty per cent.—*Med. Herald.*

